# Supplementary material for: Saturated free fatty acids and association with memory formation
Source: Nat Commun. 2021 Jun 8;12:3443. doi: 10.1038/s41467-021-23840-3 (PMC8187648; doi:10.1038/s41467-021-23840-3)
Supplement: Supplementary file 1 — Supplementary Information [file 41467_2021_23840_MOESM1_ESM.pdf]

Saturated free fatty acids and association with memory formation

Tristan P. Wallis[1#], Bharat G. Venkatesh[1#], Vinod K. Narayana[1,3#], David Kvaskoff[1,4], Alan Ho[2], Robert K. Sullivan[2], François Windels[2], Pankaj Sah[2,5] & Frédéric A. Meunier[1*]

[1]Clem Jones Centre for Ageing Dementia Research, Queensland Brain Institute, The University of Queensland, QLD 4072, Australia. [2]Queensland Brain Institute, The University of Queensland, QLD 4072, Australia. [3]Current address: Metabolomics Australia, Bio21 Institute, The University of Melbourne, VIC 3010, Australia. [4]Current address: Boehringer Ingelheim Pharma GmbH & Co. KG, Drug Discovery Sciences, Biberach an der Riß, Germany. [5]Joint Center for Neuroscience and Neural Engineering, and Department of Biology, Southern University of Science and Technology, Shenzhen, Guangdong Province, P. R. China, 518055

[#]These authors contributed equally: Tristan P. Wallis, Bharat G. Venkatesh, Vinod K. Narayana.

***Corresponding author:** Frédéric A. Meunier

e-mail: f.meunier@uq.edu.au

Supplementary Figures 1-4
Supplementary Table 1

Supplementary Figures

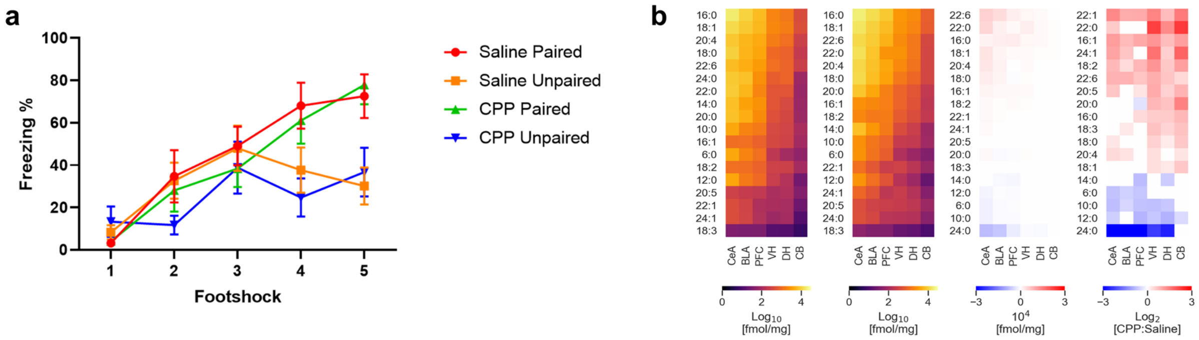

Supplementary Fig. 1 | CPP effects on acquisition of freezing response and basal FFA levels**. a,** Acquisition of auditory fear conditioning response. Average freezing response for the four experimental cohorts (Saline Paired n = 9 animals, Saline Unpaired n = 10 animals, CPP Paired n = 9 animals, CPP Unpaired n = 9 animals) after each of the five footshocks (as described in Fig. 2a). Error bars represent the standard error of the mean (SEM). CPP has no effect on the acquisition of freezing response over the course of auditory fear conditioning. **b,** Free fatty acid profile response to the NMDA receptor antagonist CPP during unpaired auditory fear conditioning stimuli. Heatmaps are vertically ordered by average intensity across 6 brain regions (CeA – central amygdala, BLA – basolateral amygdala, PFC – prefrontal cortex, VH – ventral hippocampus, DH – dorsal hippocampus, CB – cerebellum). Each pixel represents the mean FFA abundance/response from 8 animals, in a given brain region. White pixels in the fold change heatmaps represent FFAs whose change in abundance was not significant (Two-tailed *t*-test p>0.05). Source data are provided as a Source Data file.

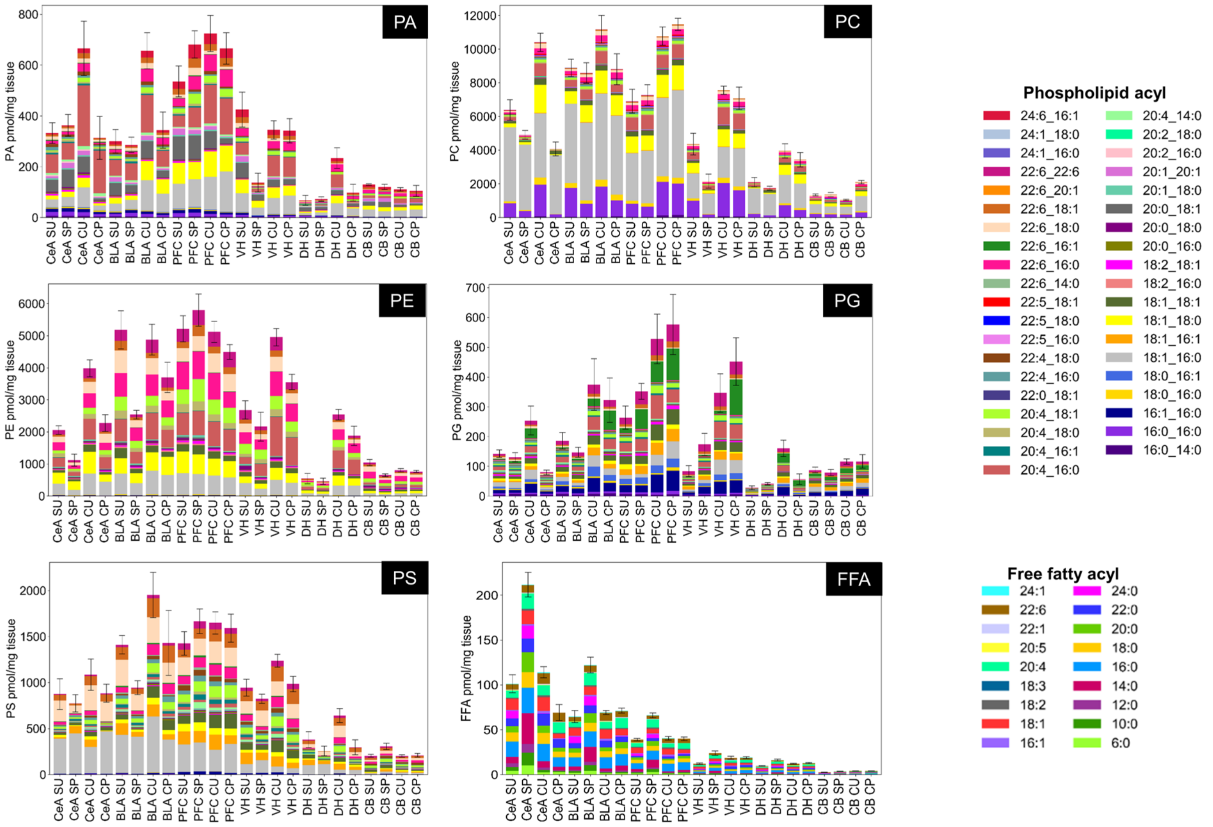

Supplementary Fig. 2 | Phospholipid and FFA profiles across brain regions and AFC experimental conditions. Bars represent the total analyte measurement for a given class, with coloured sub-bars corresponding to the mean individual analyte concentrations (pmol/mg tissue) observed across 8 animals. Error bars represent the cumulative standard error of the mean (SEM) for all analytes. Experimental conditions: SU – saline unpaired, SP – saline paired, CU – CPP unpaired, CP – CPP paired. Brain regions: CeA – central amygdala, BLA – basolateral amygdala, PFC – prefrontal cortex, VH – ventral hippocampus, DH – dorsal hippocampus, CB – cerebellum. Lipids: PA - phosphatidic acid, PC - phosphatidylcholine, PE - phosphatidylethanolamine, PG - phosphatidylglycerol, PS – phosphatidylserine. Source data are provided as a Source Data file.

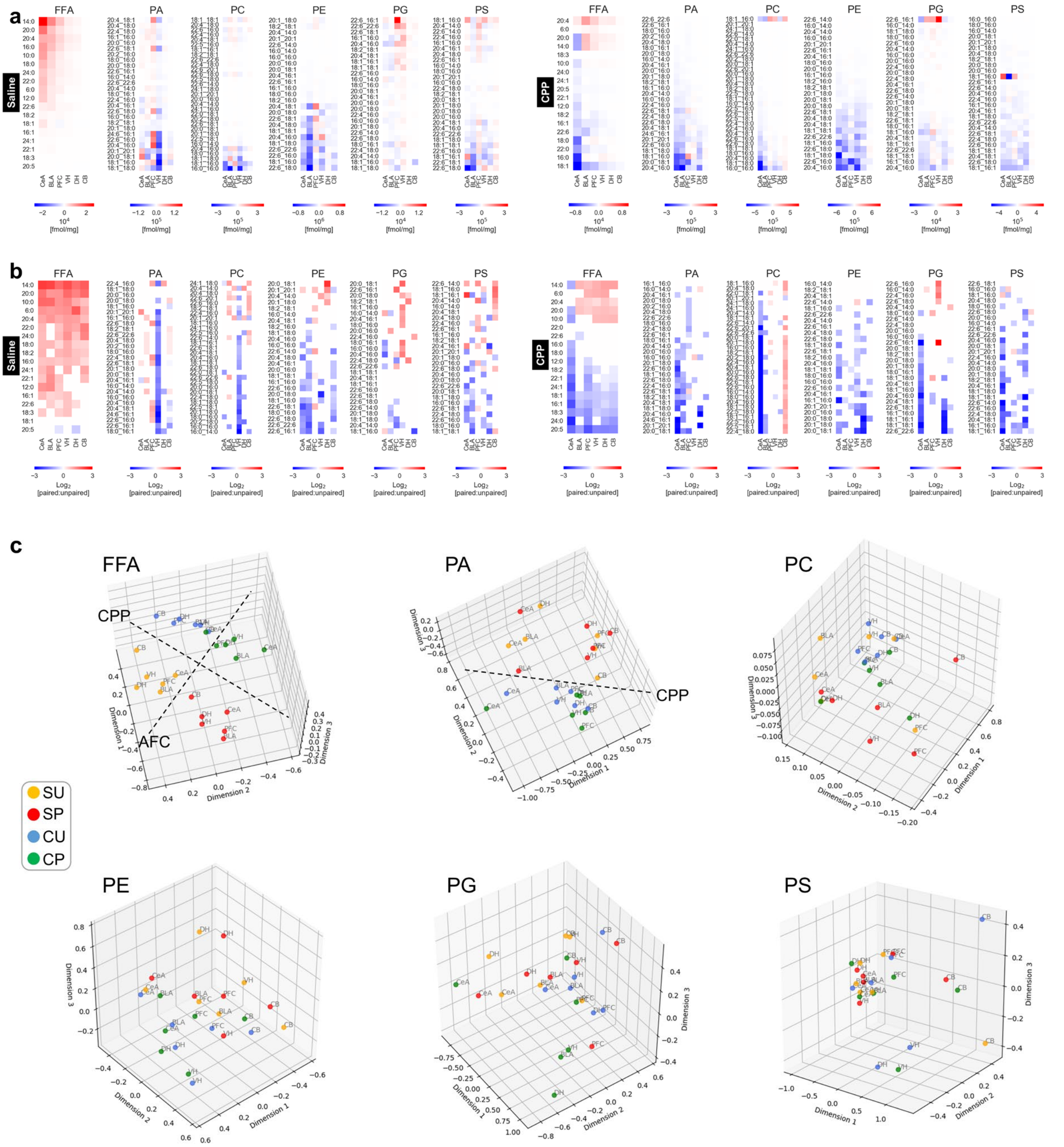

Supplementary Fig. 3 | Phospholipid and FFA responses to AFC. **a,b,** Absolute (upper panels) and fold-change (lower panels) free fatty acid and phospholipid responses to paired auditory fear conditioning stimuli in saline treated and CPP treated rats. Heatmaps are vertically ordered by average intensity across 6 brain regions. **c,** Multivariate analysis of the analyte profiles of each of the lipid classes, from 6 brain regions and 4 AFC conditions where each dot represents the normalized mean concentrations of the analytes observed across 8 animals. The isomap algorithm was used for non-linear dimensionality reduction from 18 to 3 dimensions, with N - 1 nearest neighbours being considered (where N = number of analytes). The 3D projection has been manually rotated to highlight the differences between the profiles obtained for each AFC experimental condition where possible. Dashed lines represent a manual assignment of the axes around which the profile datapoints were resolved, according to CPP treatment and AFC pairing. Experimental conditions: SU – saline unpaired, SP – saline paired, CU – CPP unpaired, CP – CPP paired. Brain regions: CeA – central amygdala, BLA – basolateral amygdala, PFC – prefrontal cortex, VH – ventral hippocampus, DH – dorsal hippocampus, CB – cerebellum. Lipids: PA -

phosphatidic acid, PC - phosphatidylcholine, PE - phosphatidylethanolamine, PG - phosphatidylglycerol, PS – phosphatidylserine. Source data are provided as a Source Data file.

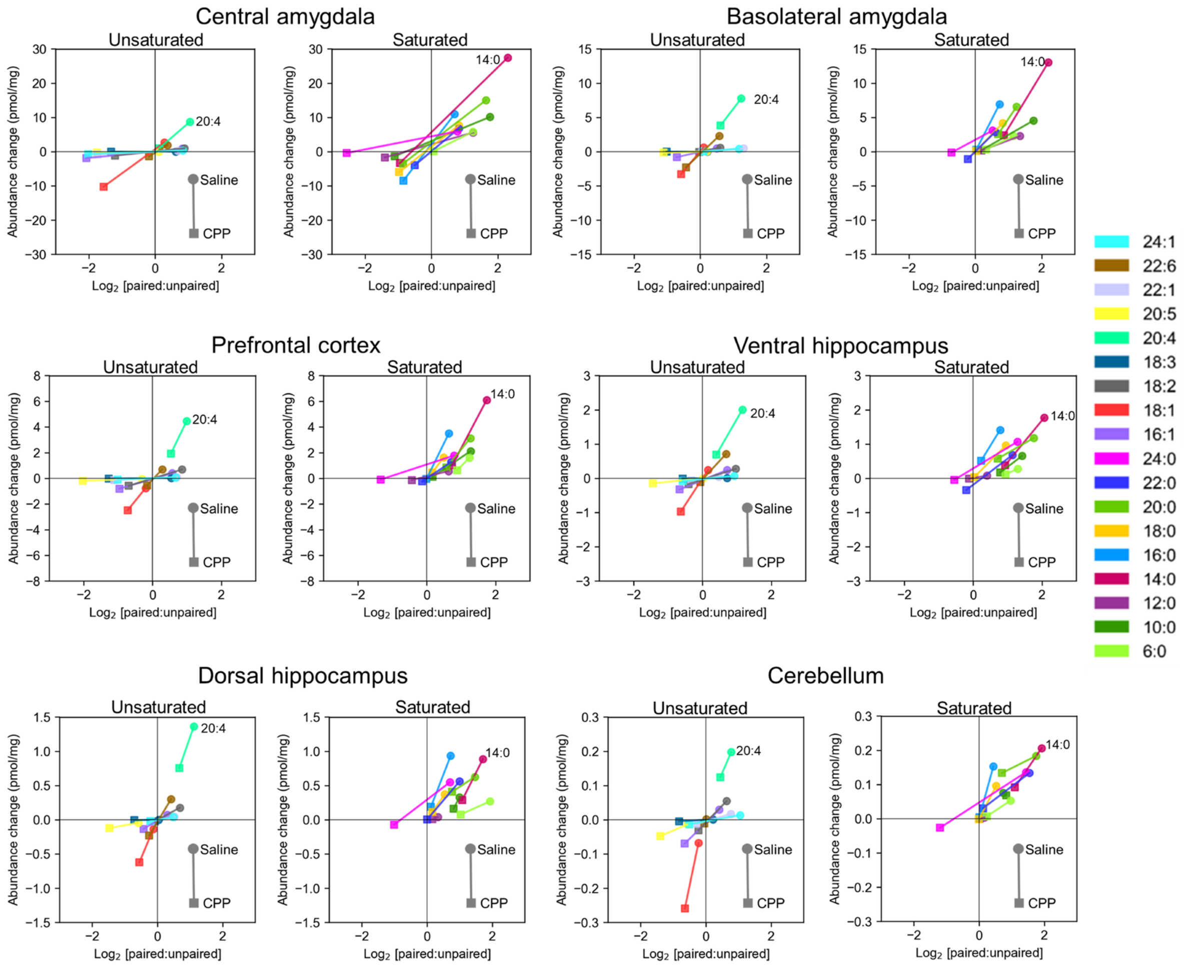

Supplementary Fig. 4 | FFA responses to paired AFC in saline- and CPP-treated rat brains. Scatterplots show the absolute change (y axis) and $\log_2$ fold change (x axis) in mean concentration for each FFA in saline (round) and CPP (square) treated animals. Arachidonic acid (20:4) and myristic acid (14:0) are indicated on each plot. Source data are provided as a Source Data file.

Supplementary Tables

Supplementary Table 1. Transitions for Multiple Reaction Monitoring

**FFA**	**MRM Q1→Q3 transitions (m/z)**		
**Species**	**FFAST-124**	**FFAST-127**	**FFAST-138**
C6:0	222.1→124.1	225.1→127.1	236.1→138.1
C10:0	278.1→124.1	281.1→127.1	292.2→138.1
C12:0	306.2→124.1	309.2→127.1	320.2→138.1
C14:0	334.3→124.1	337.3→127.1	348.3→138.1
C16:0	362.2→124.1	365.2→127.1	376.3→138.1
C16:1	360.2→124.1	363.2→127.1	374.3→138.1
C18:0	390.2→124.1	393.2→127.1	404.3→138.1
C18:1	388.3→124.1	391.3→127.1	402.3→138.1
C18:2	386.2→124.1	389.2→127.1	400.3→138.1
C18:3	384.2→124.1	387.2→127.1	398.3→138.1
C20:0	418.3→124.1	421.3→127.1	432.4→138.1
C20:4	410.2→124.1	413.2→127.1	424.3→138.1
C20:5	408.4→124.1	411.4→127.1	422.5→138.1
C22:0	446.2→124.1	449.2→127.1	460.4→138.1
C22:1	444.3→124.1	447.3→127.1	458.4→138.1
C22:6	434.2→124.1	437.2→127.1	448.3→138.1
C24:0	474.4→124.1	477.4→127.1	488.4→138.1
C24:1	472.4→124.1	475.4→127.1	486.4→138.1

**Phosphatidic acid**	**Negative (M-H)-**	
**Species**	**Q1 (m/z)**	**Q3 (m/z)**
PA 16:0_18:1	673.4	255.2
	673.4	281.2
PA 16:0_16:1	645.4	255.2
	645.4	253.2
PA 18:0_18:1	701.5	283.2
	701.5	281.2
PA 18:0_20:1	729.5	283.2
	729.5	309.2
PA 14:0_20:4	667.4	227.2
	667.4	303.2
PA 16:0_22:6	719.5	255.2
	719.5	327.1
PA 18:0_22:6	747.1	283.2
	747.1	327.1
PA 16:0_20:4	695.5	255.2
	695.5	303.2

PA 18:0_20:4	723.5	283.2
	723.5	303.2
PA 16:0_22:4	723.5	255.2
	723.5	331.2
PA 18:0_22:4	751.5	283.2
	751.5	331.2
PA 18:1_18:2	697.4	281.2
	697.4	279.2
PA 18:1_22:6	745.4	281.2
	745.4	327.1
PA 16:1_22:6	717.4	253.2
	717.4	327.1
PA 16:1_20:4	693.4	253.2
	693.4	303.2
PA 18:1_20:4	721.7	281.2
	721.7	303.2
PA 18:1_18:1	699.3	281.2
PA 16:1_18:1	671.5	253.3
	671.5	281.2
PA 20:1_20:1	755.5	309.2
PA 18:1_20:0	729.5	281.3
	729.5	311.3
PA 16:1_18:0	673.4	255.2
	673.4	283.2
PA 22:6_22:6	791.4	327.1
PA 14:0_16:0	619.4	227.1
	619.4	255.2
PA 16:0_16:0	647.4	255.2
PA 16:0_18:0	675.4	255.2
	675.4	283.2
PA 16:0_20:0	703.5	255.2
	703.5	311.2
PA 18:0_20:0	731.5	283.2
	731.5	311.2

**Phosphatidylcholine**	**Negative (M+HCOO-)**	
**Species**	**Q1 (m/z)**	**Q3 (m/z)**
PC 16:0_18:1	803.5	255.2
	803.5	281.2
PC 18:0_20:1	859.6	283.2
	859.6	309.2
PC 18:0_18:1	831.6	283.2
	831.6	281.2

PC 16:0_24:1	887.6	255.2
	887.6	365.2
PC 18:0_24:1	915.6	283.2
	915.6	365.2
PC 16:0_22:6	849.5	255.2
	849.5	327.1
PC 18:0_22:6	877.5	283.2
	877.5	327.1
PC 18:0_20:2	857.6	283.2
	857.6	307.2
PC 16:0_22:5	851.5	255.2
	851.5	329.2
PC 16:0_22:4	853.5	255.2
	853.5	331.2
PC 18:0_22:5	879.6	283.2
	879.6	329.3
PC 16:0_20:4	825.5	255.2
	825.5	303.2
PC 18:0_20:4	853.5	283.2
	853.5	303.2
PC 18:0_22:4	881.6	283.2
	881.6	331.2
PC 14:0_22:6	821.5	227.2
	821.5	327.1
PC 14:0_20:4	797.5	227.2
	797.5	303.2
PC 20:1_22:6	903.6	309.2
	903.6	327.2
PC 18:1_20:4	851.5	281.2
	851.5	303.2
PC 18:1_22:6	875.5	281.2
	875.5	327.1
PC 18:1_22:5	877.5	281.2
	877.5	329.3
PC 18:1_18:2	827.5	281.2
	827.5	279.2
PC 18:1_18:1	829.5	281.2
PC 16:1_18:1	801.5	253.3
	801.5	281.2
PC 20:1_20:1	885.6	309.2
PC 18:1_22:0	886.5	281.2
	886.5	339.3
PC 18:1_20:0	859.6	281.3

	859.6	311.3
PC 22:6_22:6	921.5	327.1
PC 16:0_16:0	777.5	255.2
PC 16:0_18:0	805.5	255.2
	805.5	283.2
PC 16:0_20:0	833.6	255.2
	833.6	311.2
PC 14:0_16:0	749.5	227.1
	749.5	255.2

**Phosphatidylethanolamine**	**Negative (M-H)-**	
**Species**	**Q1 (m/z)**	**Q3 (m/z)**
PE 16:0_18:1	716.4	255.2
	716.4	281.2
PE 16:0_16:1	688.4	255.2
	688.4	253.2
PE 18:0_18:1	744.5	283.2
	744.5	281.2
PE 18:0_20:1	772.5	283.2
	772.5	309.2
PE 14:0_22:6	734.4	227.2
	734.4	327.1
PE 14:0_20:4	710.4	227.2
	710.4	303.2
PE 16:0_22:6	762.5	255.2
	762.5	327.1
PE 18:0_22:6	790.1	283.2
	790.1	327.1
PE 18:0_20:4	738.5	283.2
	738.5	303.2
PE 16:0_20:4	766.5	255.2
	766.5	303.2
PE 18:1_18:2	740.5	281.2
	740.5	279.2
PE 18:1_22:6	788.5	281.2
	788.5	327.1
PE 16:1_22:6	760.4	255.2
	760.4	327.1
PE 16:1_20:4	736.4	255.2
	736.4	303.2
PE 18:1_20:4	764.5	281.2
	764.5	303.2
PE 18:1_18:1	732.1	281.2

PE 16:1_18:1	714.7	253.3
	714.7	281.2
PE 20:1_20:1	798.6	309.2
PE 18:1_20:0	772.5	281.3
	772.5	311.3
PE 22:6_22:6	834.5	327.1
PE 16:0_16:0	690.1	255.2
PE 16:0_18:0	718.5	255.2
	718.5	283.2
PE 14:0_16:0	662.5	227.1
	662.5	255.2
PE 16:0_20:0	746.5	255.2
	746.5	311.2
PE 18:0_20:0	774.6	283.2
	774.6	311.2

**Phosphatidylglycerol**	**Negative (M-H)-**	
**Species**	**Q1 (m/z)**	**Q3 (m/z)**
PG 16:0_18:1	747.1	255.2
	747.1	281.2
PG 16:0_16:1	719.4	255.2
	719.4	253.2
PG 18:0_18:1	775.5	283.2
	775.5	281.2
PG 18:0_20:1	803.5	283.2
	803.5	309.2
PG 14:0_22:6	765.4	227.2
	765.4	327.1
PG 14:0_20:4	741.4	227.2
	741.4	303.2
PG 16:0_22:6	793.5	255.2
	793.5	327.1
PG 18:0_22:6	821.1	283.2
	821.1	327.1
PG 16:0_20:4	769.5	255.2
	769.5	303.2
PG 16:0_22:4	797.1	255.3
	797.1	331.2
PG 18:0_22:4	825.5	283.4
	825.5	331.2
PG 18:1_18:2	771.5	281.2
	771.5	279.2
PG 18:1_22:6	819.5	281.2

	819.5	327.1
PG 16:1_22:6	791.4	255.2
	791.4	327.1
PG 16:1_20:4	767.4	253.2
	767.4	303.2
PG 18:1_20:4	795.5	281.2
	795.5	303.2
PG 18:1_18:1	773.1	281.2
PG 16:1_18:1	745.7	253.3
	745.7	281.2
PG 18:1_20:0	803.5	281.3
	803.5	311.3
PG 22:6_22:6	865.5	327.1
PG 16:0_16:0	721.1	255.2
PG 16:0_18:0	749.5	255.2
	749.5	283.2
PG 14:0_16:0	693.5	227.1
	693.5	255.2
PG 16:0_20:0	777.5	255.2
	777.5	311.2
PG 18:0_20:0	805.6	283.2
	805.6	311.2

**Phosphatidylserine**	**Negative (M-H)-**	
**Species**	**Q1 (m/z)**	**Q3 (m/z)**
PS 16:0_18:1	760.5	255.2
	760.5	281.2
PS 18:0_20:1	816.5	283.2
	816.5	309.2
PS 18:0_18:1	788.1	283.2
	788.1	281.2
PS 16:0_16:1	732.4	255.2
	732.4	253.2
PS 14:0_22:6	778.6	227.2
	778.6	327.1
PS 14:0_20:4	754.4	227.2
	754.4	303.2
PS 16:0_22:6	806.6	255.2
	806.6	327.1
PS 18:0_22:6	834.1	283.2
	834.1	327.1
PS 18:0_20:4	810.5	283.2
	810.5	303.2

PS 16:0_20:4	782.1	255.2
	782.1	303.2
PS 18:0_22:4	838.5	283.2
	838.5	331.2
PS 16:0_22:4	810.5	255.2
	810.5	331.2
PS 18:1_18:2	784.5	281.2
	784.5	279.2
PS 18:1_22:6	832.5	281.2
	832.5	327.1
PS 16:1_22:6	804.4	255.2
	804.4	327.1
PS 18:1_20:4	808.5	281.2
	808.5	303.2
PS 16:1_20:4	780.4	253.2
	780.4	303.2
PS 18:1_18:1	786.1	281.2
PS 16:1_18:1	758.5	253.3
	758.5	281.2
PS 20:1_20:1	842.5	309.2
PS 18:1_20:0	816.5	281.3
	816.5	311.3
PS 22:6_22:6	878.5	327.1
PS 16:0_16:0	734.1	255.2
PS 16:0_18:0	762.5	255.2
	762.5	283.2
PS 14:0_16:0	706.4	227.1
	706.4	255.2
PS 16:0_20:0	790.5	255.2
	790.5	311.2
PS 18:0_20:0	818.5	283.2
	818.5	311.2
