# Supplementary material for: Saturated free fatty acids and association with memory formation
Source: Nat Commun. 2021 Jun 8;12:3443. doi: 10.1038/s41467-021-23840-3 (PMC8187648; doi:10.1038/s41467-021-23840-3)
Supplement: Supplementary file 3 — Reporting Summary [file 41467_2021_23840_MOESM3_ESM.pdf]

nature research

Corresponding author(s): Fred Meunier

Last updated by author(s): May 5, 2021

Reporting Summary

Nature Research wishes to improve the reproducibility of the work that we publish. This form provides structure for consistency and transparency in reporting. For further information on Nature Research policies, see our Editorial Policies and the Editorial Policy Checklist.

Statistics

For all statistical analyses, confirm that the following items are present in the figure legend, table legend, main text, or Methods section.

n/a	Confirmed	
☐	☒	The exact sample size (*n*) for each experimental group/condition, given as a discrete number and unit of measurement
☐	☒	A statement on whether measurements were taken from distinct samples or whether the same sample was measured repeatedly
☐	☒	The statistical test(s) used AND whether they are one- or two-sided<br>*Only common tests should be described solely by name; describe more complex techniques in the Methods section.*
☒	☐	A description of all covariates tested
☐	☒	A description of any assumptions or corrections, such as tests of normality and adjustment for multiple comparisons
☐	☒	A full description of the statistical parameters including central tendency (e.g. means) or other basic estimates (e.g. regression coefficient) AND variation (e.g. standard deviation) or associated estimates of uncertainty (e.g. confidence intervals)
☐	☒	For null hypothesis testing, the test statistic (e.g. *F*, *t*, *r*) with confidence intervals, effect sizes, degrees of freedom and *P* value noted<br>*Give P values as exact values whenever suitable.*
☒	☐	For Bayesian analysis, information on the choice of priors and Markov chain Monte Carlo settings
☒	☐	For hierarchical and complex designs, identification of the appropriate level for tests and full reporting of outcomes
☒	☐	Estimates of effect sizes (e.g. Cohen's *d*, Pearson's *r*), indicating how they were calculated

Our web collection on statistics for biologists contains articles on many of the points above.

Software and code

Policy information about availability of computer code

Data collection	Lipidomics data was collected on an AB Sciex 5500 Qtrap mass spectrometer using Analyst ® 1.5.2 (AB Sciex) acquisition software. Initial quantification of analyte area under peak data was performed using Multiquant ® 3.03 (AB Sciex)
Data analysis	Auditory fear conditioning behavioural data was analysed using Prism 9.0 (Graphpad)<br>Lipidomics data was fully quantified, analysed and visualised using custom Python2.7.17 scripts using common Python modules - Scikit-learn (sklearn, 0.20.4) , Scipy (1.2.3), Numpy (1.16.6), Pandas (0.24.2) and Matplotlib (2.2.5) and Seaborn (0.9.1) . In particular, Student's t-test functionality was implemented using scipy.stats.ttest_ind, and Isomap non-linear dimensionality reduction analysis implemented using sklearn.manifold.isomap<br>All code is available upon request, and a code availability statement is included in the manuscript.

For manuscripts utilizing custom algorithms or software that are central to the research but not yet described in published literature, software must be made available to editors and reviewers. We strongly encourage code deposition in a community repository (e.g. GitHub). See the Nature Research guidelines for submitting code & software for further information.

Data

Policy information about availability of data

All manuscripts must include a data availability statement. This statement should provide the following information, where applicable:

- Accession codes, unique identifiers, or web links for publicly available datasets
- A list of figures that have associated raw data
- A description of any restrictions on data availability

The data generated during this study, presented as quantified lipid abundances, may be downloaded from the publicly accessible University of Queensland Data Collection: https://doi.org/10.14264/12793dc

Field-specific reporting

Please select the one below that is the best fit for your research. If you are not sure, read the appropriate sections before making your selection.

- [x] Life sciences
- [ ] Behavioural & social sciences
- [ ] Ecological, evolutionary & environmental sciences

For a reference copy of the document with all sections, see nature.com/documents/nr-reporting-summary-flat.pdf

Life sciences study design

All studies must disclose on these points even when the disclosure is negative.

Sample size	8 animals for each of the 4 auditory fear conditioning experimental conditions.The numbers of experimental animals used in each experimental condition (n=8) are well established in the field, and were chosen to ensure sufficient statistical power in the context of measurable behavioral responses to auditory fear conditioning.
Data exclusions	For each experimental auditory fear condition, the concentration of a given analyte was determined for 8 animals. For each set of 8 measurements, outliers were removed using a median filtering function in Python. This routine was chosen since filtering based on distance from the median works well for small datasets that are not necessarily normally distributed. Reference: T. Huang, G. Yang, and G. Tang, "A fast two-dimensional median filtering algorithm", IEEE Trans. Acoust., Speech, Signal Processing, vol. 27, no. 1, pp. 13–18, 1979
Replication	The coupling of a memory acquisition model to comparative FFA lipidomics relies on published techniques that are well established and reproducible in the respective laboratories of the participants. In particular, the FFAST lipidomics technique developed in our laboratory was has been extensively evaluated for lower limit of quantification, accuracy and inter assay reproducibility. Narayana, V. K., Tomatis, V. M., Wang, T., Kvaskoff, D. & Meunier, F. A. Profiling of Free Fatty Acids Using Stable Isotope Tagging Uncovers a Role for Saturated Fatty Acids in Neuroexocytosis. Chem Biol 22, 1552-1561, doi:10.1016/j.chembiol.2015.09.010 (2015).
Randomization	For each of the four auditory fear conditioning experimental conditions, rats were randomly selected from a larger cohort of 6-8 week old male animals.
Blinding	The experimental design of this study is based on all animals being treated identically pre- and post-conditioning, such that differences in lipid concentrations in the animals are maximally due to response to the treatment conditions. Each animal was assigned an internal ID, and the subsequent brain excision, FFA extraction and LCMS data acquisition/quantification were performed using only the ID, such that the investigators were blind to the experimental condition.

Reporting for specific materials, systems and methods

We require information from authors about some types of materials, experimental systems and methods used in many studies. Here, indicate whether each material, system or method listed is relevant to your study. If you are not sure if a list item applies to your research, read the appropriate section before selecting a response.

Materials & experimental systems

n/a	Involved in the study	
[ ]	[ ]	Antibodies
[x]	[ ]	Eukaryotic cell lines
[x]	[ ]	Palaeontology and archaeology
[ ]	[x]	Animals and other organisms
[x]	[ ]	Human research participants
[x]	[ ]	Clinical data
[x]	[ ]	Dual use research of concern

Methods

n/a	Involved in the study	
[x]	[ ]	ChIP-seq
[x]	[ ]	Flow cytometry
[x]	[ ]	MRI-based neuroimaging

Antibodies

Antibodies used	No antibodies were used in this study
Validation	No validation of antibodies was required for this study

Animals and other organisms

Policy information about studies involving animals; ARRIVE guidelines recommended for reporting animal research

Laboratory animals	Male Sprague Dawley rats, 6-8 weeks old

Wild animals	No wild animals were used in this study
Field-collected samples	No field collected samples were used in this study
Ethics oversight	In all procedures, the care and experimental use of animals was in accordance with protocols approved by the University of Queensland Animal Ethics Committee (QBI/313/13/NHMRC).<br>This statement is included in the manuscript

Note that full information on the approval of the study protocol must also be provided in the manuscript.
